# Impact of Dietary Fiber on West Nile Virus Infection

**DOI:** 10.3389/fimmu.2022.784486

**Published:** 2022-02-28

**Authors:** Duan Ni, Jian Tan, Paula Niewold, Alanna Gabrielle Spiteri, Gabriela Veronica Pinget, Dragana Stanley, Nicholas Jonathan Cole King, Laurence Macia

**Affiliations:** ^1^ Charles Perkins Centre, The University of Sydney, Sydney, NSW, Australia; ^2^ School of Medical Sciences, Faculty of Medicine and Health, The University of Sydney, Sydney, NSW, Australia; ^3^ Department of Infectious Diseases, Leiden University Medical Centre, Leiden, Netherlands; ^4^ School of Health, Medical and Applied Science, Central Queensland University, Rockhampton, QLD, Australia; ^5^ Sydney Institute for Infectious Diseases, The University of Sydney, Sydney, NSW, Australia; ^6^ Sydney Cytometry, The University of Sydney and The Centenary Institute, Sydney, NSW, Australia

**Keywords:** dietary fiber, gut microbiota, West Nile Virus (WNV), infection, immune response, enteric neurons, cytokines

## Abstract

Dietary fiber supports healthy gut bacteria and their production of short-chain fatty acids (SCFA), which promote anti-inflammatory cell development, in particular, regulatory T cells. It is thus beneficial in many diseases, including influenza infection. While disruption of the gut microbiota by antibiotic treatment aggravates West Nile Virus (WNV) disease, whether dietary fiber is beneficial is unknown. WNV is a widely-distributed neurotropic flavivirus that recruits inflammatory monocytes into the brain, causing life-threatening encephalitis. To investigate the impact of dietary fiber on WNV encephalitis, mice were fed on diets deficient or enriched with dietary fiber for two weeks prior to inoculation with WNV. To induce encephalitis, mice were inoculated intranasally with WNV and maintained on these diets. Despite increased fecal SCFA acetate and changes in gut microbiota composition, dietary fiber did not affect clinical scores, leukocyte infiltration into the brain, or survival. After the brain, highest virus loads were measured in the colon in neurons of the submucosal and myenteric plexuses. Associated with this, there was disrupted gut homeostasis, with shorter colon length and higher local inflammatory cytokine levels, which were not affected by dietary fiber. Thus, fiber supplementation is not effective in WNV encephalitis.

## Introduction

Consumption of dietary fiber confers health benefits and correlates with decreased mortality from both infectious and non-infectious diseases (1). Dietary fiber comprises non-digestible complex carbohydrates that promote gut health through the beneficial reshaping of the gut microbiota. This occurs through the release of bacterial metabolites, particularly short-chain fatty acids (SCFA), during its fermentation in the colon (2). High-fiber feeding and SCFA reduce disease severity in models of colorectal cancer, colitis and food allergy (3, 4). The benefits of dietary fiber and SCFA extend beyond the gastrointestinal tract, influencing immune responses in the lungs in a model of allergic airway inflammation (5), in the pancreas in a model of type 1 diabetes (6) or in the brain in multiple sclerosis (7). Dietary fiber and SCFA are also beneficial in infectious diseases with decreased mortality in a mouse model of influenza infection (8) and of *Citrobacter rodentium* infection (9).

Dietary fiber and SCFA have a broad impact on the immune system by promoting the development of anti-inflammatory regulatory T cells (10) and regulatory B cells (11), of Th1 cells (12), of memory CD8^+^ T cells (13, 14) and by increasing B cell antibody production (15). They also affect innate immune cells by modulating the migration and activation of neutrophils (16, 17), the generation of monocytes and their differentiation into anti-inflammatory macrophages (8), the production of IL-22 by type 3 innate lymphoid cells (18) and the activity of CD103^+^ dendritic cells (4). The mechanisms behind the immunomodulatory effects of SCFA are multifaceted. They occur *via* activation of G-protein-coupled receptors, through modulation of gene expression by inhibiting histone deacetylases, by affecting immune cell metabolic activity by fueling the tricarboxylic acid cycle, or by promoting glutaminolysis, fatty acid oxidation and gluconeogenesis (2, 19).

Antibiotic treatments disrupt both the gut microbiota and its release of SCFA (20), impairing immunity, including the IFN-γ antiviral immune response, aggravating influenza severity (21). Similarly, antibiotic treatment exacerbates disease severity in a model of West Nile Virus (WNV) infection (22), suggesting that the gut microbiota can regulate WNV, infection outcomes.

WNV is a mosquito-borne neurotropic flavivirus. Neuronal infection is associated with massive inflammatory monocyte recruitment into the central nervous system (CNS), causing life-threatening encephalitis (23–26). As dietary fiber promotes anti-inflammatory SCFA production and has been shown to be protective in a model of influenza virus infection by targeting monocytes and enhancing CD8^+^ T cell effector function (8), we hypothesized that high-fiber feeding might also be protective in a mouse model of WNV encephalitis.

To test this hypothesis, we fed mice on diets abundant or deficient in dietary fiber, prior to infection with either 100% or 50% lethal doses (LD_100_ or LD_50_) of WNV and investigated the impact of these diets on survival and immune profile.

## Materials and Methods

### Mice and Dietary Intervention

Six-week-old female C57BL/6 mice [Animal BioResource (NSW, Australia) or Animal Resources Centre (WA, Australia)] were housed under specific pathogen-free conditions in the animal facility of the Charles Perkins Centre. Diets were purchased from Specialty Feeds (Glenn Forest, Australia) and mice were fed for 2 weeks prior to infection, either with commercially available diets deficient in dietary fiber (SF11-028), or enriched in dietary fiber (SF11-025: resistant starch gel crisp as source of fiber) as previously described (3). Experiments were performed in accordance with the animal ethics protocol 2016/976 approved by The University of Sydney Animal Ethics Committee.

### WNV Infection

The original stock of WNV (lineage II Sarafend strain), acquired from The John Curtin School of Medical Research (ACT, Australia) was propagated alternately in C57BL/6 suckling mouse brains and *in vitro* in Vero cells (24). Mice anesthetized intraperitoneally with Avertin at day 0 were inoculated intranasally with 6 × 10^4^ and 6 × 10^3^ plaque-forming units (PFUs) of WNV to achieve 100% lethal dose (LD_100_) and 50% lethal dose (LD_50_) studies, respectively, as described in (27). Alternatively, mice were injected with a LD_100_ intracranially *via* the postglenoid foramen, an approach that does not penetrate the skull bones, minimizing tissue damage (28). Mice were weighed daily, and assessed for clinical symptoms, as previously described (24). Briefly, they were scored as follows: score 0: no clinical signs, 1: Weight loss < 5%, 2: Weight loss ≥ 5%, 3: Weight loss ≥ 5% with significant reduction in movement, 4: Weight loss ≥ 5% with significant reduction in movement, ruffled fur, hunched posture, and seizures and 5: Immobile, cold.

### Flow Cytometry and Data Analysis

Mice were anesthetized and perfused with ice-cold PBS before collection and processing of tissues into single cell suspension as previously described (26, 29). Dead cells were excluded, based on their staining with the LIVE/DEAD™ Fixable Blue Dead Cell Stain (ThermoFisher Scientific) and anti-mouse CD16/32 (BioLegend) was used to prevent antibody non-specific binding. Cells were permeabilized with the Cytofix/Cytoperm kit (BD Biosciences) for intracellular staining. Antibodies used for flow cytometry are listed in **Table S1**. To study cytokine expression, cells were stimulated with 50ng/ml phorbol 12-myristate 13-acetate, 500ng/ml ionomycin and 5µg/ml brefeldin A for 4 hours. Flow cytometry was run on the LSR-II analyser (Becton Dickinson, San Jose, CA, USA) using the FACSDiva software and data was analyzed with FlowJo v10.7.1. (Treestar Inc. Ashland, OR, USA) based on gating strategies presented in **Figures S2-3**.

### RNA Extraction and Quantitative Real-Time PCR

Total tissue RNA was extracted using TRI Reagent (Sigma Aldrich), based on the manufacturer’s protocol. cDNA was synthesized with the High-Capacity cDNA Reverse Transcription Kit (ThermoFisher Scientific). qPCR was conducted with the Power SYBR™ Green PCR Master Mix (ThermoFisher Scientific) on LightCycle^®^ 480 Instrument II (Roche) with primers listed in **Table S2**.

### Histology

Paraformaldehyde-fixed (4% in PBS), paraffin-embedded colon tissues were sectioned and stained with H&E and imaged using light microscopy (Zeiss Axioscope, Zeiss, Oberkochen, Germany). Colonic inflammation was scored, based on previously published guidelines (30).

### Immunohistochemistry/Immunofluorescence

For immunohistochemistry, colon tissue was fixed overnight in 2% PFA and subsequently placed in a series of solutions of progressively increasing sucrose concentration (10%, 20% and 30% sucrose in PBS), before being embedded in optimum cutting temperature compound (O.C.T.; Tissue-Tek) and frozen in hexane pre-chilled in liquid nitrogen, as previously described (26, 31). Tissue blocks were sectioned (8–9 µm), fixed in methanol, rinsed in tris-buffered saline with 0.05% Tween 20 (TBST) and blocked with 10% FCS before being stained with primary fluorophore-conjugated antibodies targeting WNV non-structural protein 1 (NS-1) and FOX-3 in neuronal nuclei (NeuN). Tissue sections were washed twice in TBST before being counterstained with DAPI antifade (Vector Laboratories). Images were acquired on the Olympus BX51 Microscope using a DP-70 camera and Cell Sensor software.

### Acetate Quantification

Fecal SCFA acetate was quantified by ^1^H nuclear magnetic resonance spectroscopy as previously described (11). Feces were first homogenized with deuterium oxide (Sigma-Aldrich) at a concentration of 100mg/ml and centrifuged for 5 minutes at 14000 × g at 4°C. The resulting supernatant was then diluted in sodium triphosphate buffer (pH=7.0) (Sigma-Aldrich), with 0.5mM 4,4-dimethyl-4-silapentane-1-sulfonic acid as the internal standard (Sigma-Aldrich). Samples were run on Bruker 600 MHz AVANCE III Spectrometer and analyzed with the Chenomx NMR Suite v8.4 (Chenomx Inc.).

### 16S rRNA Gene Sequencing

DNA from fecal samples were extracted using the FastDNA Spin Kit for Feces (MP Biomedicals) following the manufacturer’s protocol. The primers for 16S microbiota analysis were selected to amplify the V3-V4 region of 16S rRNA genes. Forward primer used was ACTCCTACGGGAGGCAGCAG and reverse GGACTACHVGGGTWTCTAAT. The primers contained barcodes, spacers and Illumina sequencing linkers and were designed and used as suggested by Fardosh et al. (32). The 16S rRNA gene sequencing library preparation, PCR amplification and library purification followed the Illumina recommended protocol (Illumina Inc., San Diego, CA, USA). The sequencing was performed on an Illumina MiSeq instrument using 2x300 bp paired-end sequencing. Amplicon sequence variant was generated with the dada2 package (1.16.0) using R software (4.0.2). Taxonomy was assigned using the Ribosomal Database Project classifier with species level taxonomy assignment. Alpha and beta diversity analysis was performed using the phyloseq (1.32.0), microbiome (1.10.0) and vegan (2.5-7) packages. Sequence data was deposited in the European Nucleotide Archive under accession number PRJEB50194.

### Statistics

Unpaired t-test and ANOVA were used to compare two or more groups, respectively, and Mantel-Cox log-rank test was used to analyze LD_50_ study survival results. Differences were considered statistically significant when p<0.05.

## Results

### High-Fiber Feeding Does Not Affect WNV-Induced Encephalitis Neuroinflammation

**Figure 1:**
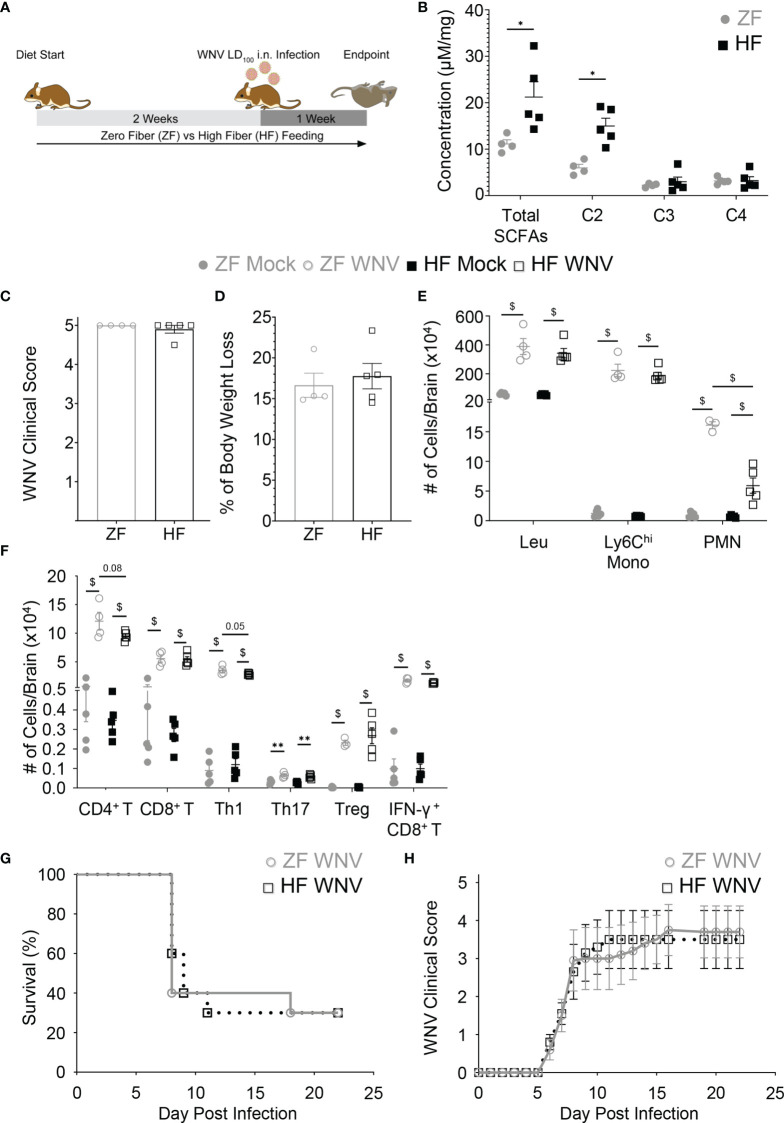
Dietary fiber increased fecal acetate but failed to protect against WNV encephalitic neuroinflammation and improve disease survival. Mice were fed on diets either enriched (HF) or deficient (ZF) in dietary fiber for two weeks and then intranasally infected with either LD_100_ **(A–F)** or LD_50_ WNV **(G, H)**, while diets were maintained during infection. **(A)** Experimental workflow for LD_100_ WNV infection study. **(B)** The concentration of fecal total SCFA, acetate (C2), propionate (C3) and butyrate (C4) of mice fed on HF or ZF diet for two weeks was quantified by NMR spectroscopy (n = 4-5 per group). Clinical scores **(C)** and body weight loss **(D)** of ZF- or HF-diet-fed mice in WNV LD_100_ study were determined at 7 dpi (n = 4-5 per group). **(E)** Numbers of total leukocytes (Leu), Ly6C^hi^ monocytes (Ly6C^hi^ Mono), and neutrophils (PMN) in mock-infected (Mock) or 7 dpi LD_100_ WNV-infected (WNV) brain from mice fed on ZF- or HF-diets were analyzed by flow cytometry (n = 4-5 per group). **(F)** Numbers of CD4^+^ T cells, CD8^+^ T cells, Th1, Th17, Treg, and IFN-γ^+^ CD8^+^ T cells in mock-infected (Mock) or 7 dpi LD_100_ WNV-infected (WNV) brain from mice fed on ZF- or HF-diets were analyzed by flow cytometry (n = 4-5 per group). **(G)** Survival of mice intranasally infected with LD_50_ WNV fed on ZF- or HF-diets (n = 10 per group). **(H)** Average clinical scores of LD_50_-infected mice fed on ZF- or HF-diets (n = 10 per group). Data are represented as mean ± SEM with ^∗^p < 0.05; ^∗∗^p < 0.01 and ^$^p < 0.001 by t test or two-way ANOVA.

To determine whether beneficial reshaping of the gut microbiota could affect the severity of WNV infection, mice were fed on diets enriched in dietary fiber (high fiber, HF) or deficient in dietary fiber (zero fiber, ZF) for 2 weeks prior to infection (**Figure 1A**). These diets have previously been shown to reshape the gut microbiota composition beneficially (HF) or detrimentally (ZF) (3, 4). Mice fed on HF, as expected, had a significantly different gut microbiota composition as shown by weighted UniFrac (**Figure S1**), as well as a significantly increased production of the SCFA, acetate, in feces, compared to ZF-fed mice (**Figure 1B**), as previously published (5). These mice were inoculated intranasally with WNV LD_100_ and maintained on these diets over the course of infection. Intranasal inoculation enables reliable infection of the central nervous system (CNS), with clear separation of anti-viral responses in the CNS from systemic responses. In this model, WNV spreads from the olfactory bulb to the rest of the brain and spinal cord over the course of infection and has been described in detail by Getts et al. (26).

Both HF- and ZF-fed mice had altered gut microbiota composition after WNV infection (**Figures S1A–G**), with the bacteria from the genus *Dorea, Enterorhabdus* and *Clostidium IV* being significantly more represented in infected ZF-fed mice. Despite these differences, acetate levels remained higher in infected HF-fed mice (**Figure S1H**). Nevertheless, both groups had similar clinical scores at 7 days post infection (dpi) (**Figure 1C**), as well as undergoing comparable weight loss (**Figure 1D**). This suggests that HF diet does not protect against LD_100_ WNV infection. Consistent with this, total numbers of leukocytes in the brain and infiltration of inflammatory Ly6C^hi^ monocytes was similar in both groups, as assessed by flow cytometry, although neutrophil infiltration was significantly reduced in HF-fed mice (**Figure 1E** and **Figures S2, S3**). The aggravating effects of antibiotics have been linked to impaired WNV induced-T cell responses (22). In contrast, HF and SCFA can modulate T cell differentiation towards both Th1, Th17 and Treg, depending on the context (12). However, numbers of infiltrating CD4^+^ and CD8^+^ T cells into the brain were similar between HF and ZF groups (**Figure 1F**). Furthermore, in WNV infection, HF feeding did not affect the T cell response, with similar numbers of Th1, Th17 and Treg, as well as IFN-γ^+^ CD8^+^ T cells, both in the brain (**Figure 1F**) and its draining lymph nodes, when compared to ZF (**Figure S4**).

As beneficial immunomodulation by HF diet may be masked by overwhelming infection at the LD_100_, we investigated the effects of dietary fiber in WNV infection at LD_50_ and assessed its impact on survival. In mice inoculated intranasally with LD_50_ as in **Figure 1A**, clinical disease scores increased to a similar extent in both dietary interventions from 5 dpi onwards (**Figure 1H**). By 10 dpi, independent of diet, the mortality of both groups was similar at 30-50% and remained stable after 16 dpi (**Figure 1G**). We confirmed that the absence of protection in the HF group was not linked to this diet in particular, as a different high-fiber diet enriched in guar gum, previously shown to improve health (3–5), also had no impact (data not shown). Taken together, these data indicate that dietary fiber does not reduce the clinical severity of WNV encephalitis.

### WNV Encephalitis Is Associated With the Spread of the Virus to the Colon

**Figure 2:**
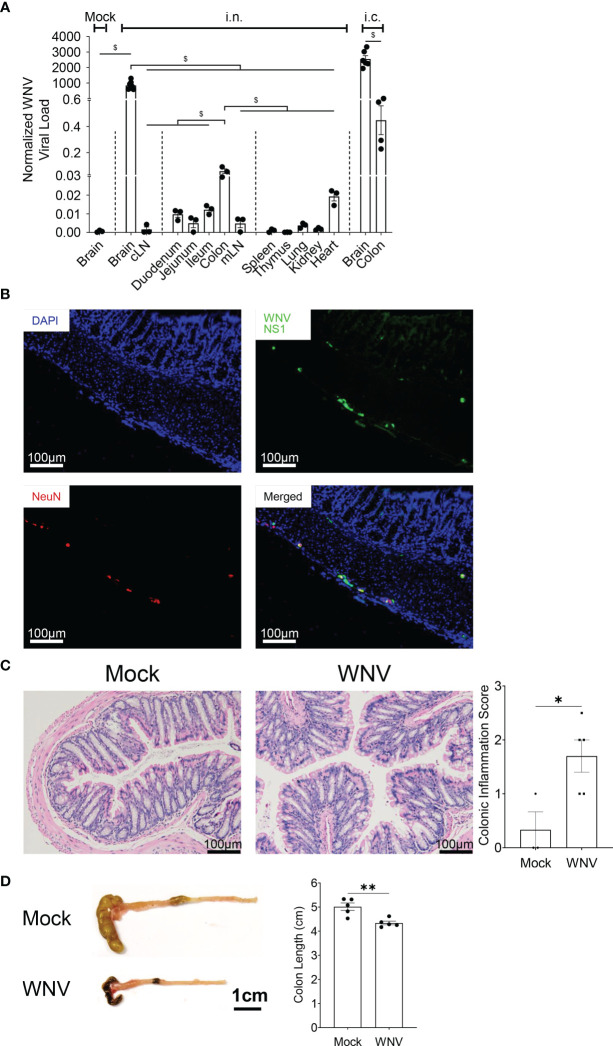
Spreading of WNV in the colon during WNV encephalitis triggers colonic inflammation. Mice were fed on normal chow diet and were intranasally (i.n.) or intracranially (i.c.) infected with LD_100_ WNV. **(A)** WNV viral load quantified for mock-infected animals (Mock) or infected animal at 7dpi by qPCR in brain, cLN, duodenum, jejunum, ileum, colon, mLN, spleen, thymus, lung, kidney, heart, n = 3-7 per organ), as well as brain and colon from mice inoculated intracranially with LD_100_ WNV, as a control. **(B)** Representative immunofluorescence staining of WNV non-structural protein 1 (WNV NS1, green), and FOX-3 neuronal nuclei (NeuN, red) in colon, counterstained with DAPI (blue) from 7 dpi LD_100_ WNV-infected mice fed on a normal diet. Scale bar represents 100μm. **(C)** H&E-stained colonic tissue sections from mock-infected (Mock) or 7 dpi LD_100_ WNV-infected (WNV) mice fed on a normal diet evaluated for colonic inflammation. Representative histological images are shown for each group in the left panel, and quantification of colonic inflammation scoring are shown in the right panel (n = 3-5 per group). Scale bar represents 100μm. **(D)** Colon length of mock-infected (Mock) or 7 dpi LD_100_ WNV-infected (WNV) mice fed on normal diet were assessed (n = 5 per group). Scale bar represents 1cm. Data are represented as mean ± SEM with ^∗^p < 0.05; ^∗∗^p < 0.01; ^$^p < 0.001 by t test or two-way ANOVA.

Systemic infection associated with peripheral WNV inoculation has been linked to enteric neuronal infection and neuronal injury promoted by antigen-specific CD8^+^ T cells (33), resulting in altered gut homeostasis, in particular, gastrointestinal motility (33). Whether CNS infection with WNV is also associated with WNV in the gastrointestinal tract is unknown. To address this question, we inoculated mice fed on normal chow diet intranasally (i.n.) with LD_100_ WNV and assessed the presence of WNV by qPCR in the brain and brain-draining cervical lymph nodes (cLN), the gastrointestinal tract (duodenum, jejunum, ileum and colon), the gut-draining mesenteric lymph nodes (mLN), the primary lymphoid organs (thymus and spleen) and peripheral organs (lung, kidney and heart). While the brain was the major site of infection, we also detected significant viral load in the colon and at lower levels in the duodenum, jejunum, ileum and their mLN, as well as the heart. Viral load in the colon was even higher in animals inoculated intracranially (i.c.) with WNV, excluding the possibility that infection was a result of systemic infection or ingestion (**Figure 2A**). We confirmed the presence of WNV by immunofluorescence in the colon, specifically in colonic neurons of the myenteric and submucosal plexuses, colocalizing with the neural marker NeuN (**Figure 2B**). We then assessed whether the presence of WNV was associated with colonic mucosal inflammation. Histologically, mice infected with WNV had significantly more mononuclear cells infiltrating the colon (**Figure 2C**), and this was associated with a significantly reduced colon length in infected mice (**Figure 2D**), as well as changes in the gut microbiota composition (**Figure S5**). Thus, CNS infection with WNV is accompanied by spread of virus in the gastrointestinal tract, particularly in colonic neurons, leading to local inflammation.

### Dietary Fiber Does Not Affect Colonic Viral Load and Minimally Affects Colonic Inflammation

**Figure 3:**
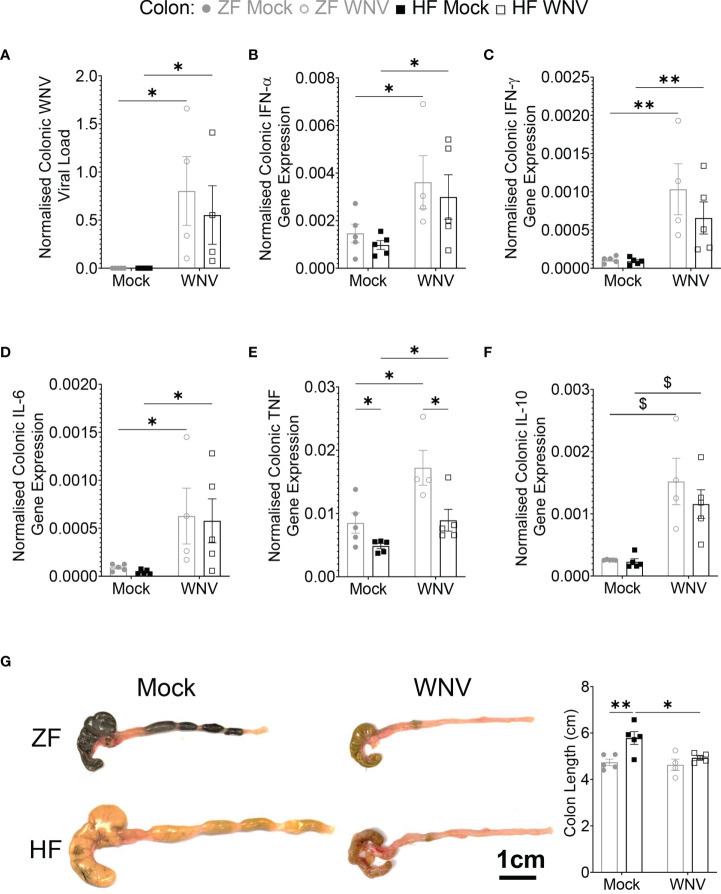
Dietary fiber did not ameliorate WNV-induced colonic inflammation despite reducing TNF. Mice were fed on diets either enriched (HF) or deficient (ZF) in dietary fiber for two weeks and then intranasally infected with LD_100_ WNV. WNV viral load **(A)**, colonic gene expression of *Ifna* **(B)**, *Ifng* **(C)**, *Il6* **(D)**, *Tnf* **(E)**, *Il10* **(F)** was determined by qPCR (n = 4-5 per group). **(G)** Colon length of mock-infected (Mock) or 7 dpi LD_100_ WNV-infected (WNV) mice fed on ZF- or HF-diets were assessed (n = 4-5 per group). Scale bar represents 1cm. Data are represented as mean ± SEM with ^∗^p < 0.05; ^∗∗^p < 0.01; ^$^p < 0.001 by two-way ANOVA.

As dietary fiber is known to promote gut health, we then investigated whether HF affected viral load in the colon. Regardless of diet, mice fed on HF or ZF had similar colonic viral load (**Figure 3A**), showing no effect of dietary fiber. The presence of virus was associated with significantly increased mRNA expression for type 1 and type 2 IFNs, IL-6, tumor necrosis factor (TNF) and IL-10 expression in the colon in both groups (**Figures 3B–F**). While levels of mRNA for these cytokines were similar between groups, mice fed on HF had significantly decreased levels for TNF, compared to mice fed on ZF, suggesting potential local immunomodulatory effects of HF in the colon (**Figure 3E**). The similar overall levels of message for cytokines (except for TNF) correlated with similar increased mononuclear cell infiltration by histological analysis between groups (data not shown). Infected HF-fed mice also had immune phenotypic profiles in the mLN similar to infected ZF-fed mice (**Figure S6**), suggesting a similar response to infection in both groups.

The fermentation of dietary fiber affects both the colon and cecum length (3). Accordingly, the colons of mock-infected HF-fed mice were significantly longer than ZF-fed mice. However, in WNV infection by 7 dpi, colon length in HF-fed mice was significantly reduced and comparable in size to those of ZF-fed mice. Notably, the colon length in infected ZF-fed mice was similar to mock-infected ZF-fed mice (**Figure 3G**).

Together, these data show that WNV infection of the CNS is accompanied by infection of the autonomic nervous plexuses in the colon, mucosal mononuclear cellular infiltration, local elevation of immune cytokines and reduced colonic size, consistent with inflammation, which, despite reduced TNF levels, was unaffected by the fiber content in HF feeding.

## Discussion

In the present report, we show that dietary intervention modulating dietary fiber had no benefit in WNV encephalitic disease, notwithstanding an evident shift in the composition of the gut microbiota. Mice fed on a diet enriched or deficient in dietary fiber showed similar degrees of encephalitis, as evident by their clinical scores and mortality rates, and except for neutrophils, immune cell recruitment into the brain and draining lymph nodes was similar in both groups. We used an intranasal or intracranial inoculation model of WNV infection to limit systemic infection, detecting high levels of WNV in the brain, as expected. However, significant virus signal was also detected in the colon, principally in neurons, accompanied by a local mucosal inflammatory infiltrate. Supporting this, mRNA for both pro-and anti-inflammatory cytokines, as well as type 1 and 2 IFNs were increased locally and there was a marked reduction in colonic length, a feature of colonic inflammation. Consistent with HF treatment in other inflammatory diseases, TNF message was reduced in HF-fed mice, however, no other measured parameters were altered by high-fiber content in the diet.

Dietary fiber and SCFA have been shown to have immunomodulatory effects beneficial in lung infection (8) and in inflammatory CNS disease (7). Since detrimental changes in the gut microbiota induced by antibiotics were reported to aggravate flavivirus infection (22), we hypothesized that high-fiber diet may ameliorate disease in WNV encephalitis. However, in our experiments, neither the type of fiber, nor the amount was beneficial in WNV infection. Using two different types of diet, one supplemented with guar gum, containing the equivalent of 10-fold the recommended amount of fiber (3), we found no benefit, either in clinical scores or increased survival. Thus, increasing the consumption of dietary fiber in itself is unlikely to be an effective treatment in WNV encephalitis, as the immunomodulatory effects of dietary fiber are evidently insufficient to counteract the damaging immunopathology associated with the anti-viral response. Nevertheless, high fiber may potentiate the effects of anti-inflammatory drugs, which to our knowledge has never been investigated.

The only change we observed in the brains of HF-fed mice with WNV encephalitis was decreased neutrophilic infiltration. A similar effect of HF and acetate was reported in a mouse model of gout in which HF-fed mice had reduced neutrophilic inflammation (34). Acetate increased neutrophil apoptosis, which decreased the inflammation and promoted resolution in the knee. The role of neutrophils in WNV is unclear and may either promote virus replication or contribute to disease resolution (35). However, in our study the potential impact of HF on neutrophil recruitment or apoptosis did not improve survival, suggesting a minor role of these cells, as supported by our previous studies (25). This may explain why dietary fiber may be beneficial in influenza infection (8) in which neutrophils contribute significantly to immunopathology, but not in WNV.

We used intranasal or intracranial infection to limit systemic infection. In both cases, WNV was also detectable in the colon, evidently localized to neurons of the myenteric and submucosal plexuses, as well as to a lesser extent in several other organs. The gut-brain connection has been reported in other infection models, with spread of reovirus from the intestine to the brain *via* the vagus nerve, for example (36). WNV may reach colonic neurons from the CNS *via* efferent branches of the vagus nerve. This would explain the presence of the virus in the heart, as well as other viscera supplied by the extensive parasympathetic network originating from this important cranial nerve, while the well-described interconnectivity between enteric and mucosal plexuses, would explain the presence of virus at both levels (37). The presence of WNV in the colon correlated with local mucosal inflammation characterized by increased levels of IFN-⍺ and IFN-γ, as well as IL-6 and TNF, although the latter was reduced in HF-fed mice. While reduced TNF expression is consistent with the immunomodulatory effects of HF reported in colitis, it is unclear why levels of IL-6 and IL-10 are not similarly affected. The presence of WNV in the gastrointestinal tract has been reported to impair gut motility (33) in a systemic infection model. It is thus of interest that direct brain infection can also be relayed to the colon, where it presumably contributes to this dysmotility by interfering with vagal stimulation, which in itself may interfere with the beneficial effect of HF feeding seen in non-infectious models of colitis.

Thus, despite its potent immunomodulation and palliative success in other inflammatory diseases, the use of high-fiber feeding is not an effective treatment in WNV infection.

## Data Availability Statement

The raw data supporting the conclusions of this article will be made available by the authors, without undue reservation.

## Ethics Statement

The animal study was reviewed and approved by The University of Sydney Animal Ethics Committee.

## Author Contributions

DN and JT performed most of the experiments and analysis and wrote the manuscript. PN and GP did the flow cytometry experiments related to brains and lymph nodes and contributed to manuscript writing. AS did the immunofluorescence staining experiments and contributed to immune analysis and manuscript writing. DS helped with the 16S sequencing and analysis. NK and LM conceived, designed, and supervised the study, participated in the experiments and wrote the manuscript. All authors reviewed the manuscript. All authors contributed to the article and approved the submitted version.

## Funding

This project was funded by the Australian Research Council grant LPP160100627. DN is a recipient of the Australian Government Research Training Program Scholarship (International). AGS is a recipient of the Australian Government Research Training Program Scholarship and The University of Sydney Postgraduate Merit Award. LM is an a’Beckett fellow.

## Conflict of Interest

The authors declare that the research was conducted in the absence of any commercial or financial relationships that could be construed as a potential conflict of interest.

## Publisher’s Note

All claims expressed in this article are solely those of the authors and do not necessarily represent those of their affiliated organizations, or those of the publisher, the editors and the reviewers. Any product that may be evaluated in this article, or claim that may be made by its manufacturer, is not guaranteed or endorsed by the publisher.
