# Supplementary material for: Impact of Dietary Fiber on West Nile Virus Infection
Source: Front Immunol. 2022 Feb 28;13:784486. doi: 10.3389/fimmu.2022.784486 (PMC8919037; doi:10.3389/fimmu.2022.784486)
Supplement: Supplementary file 1 [file DataSheet_1.pdf]

SUPPLEMENTARY FIGURE 1-1

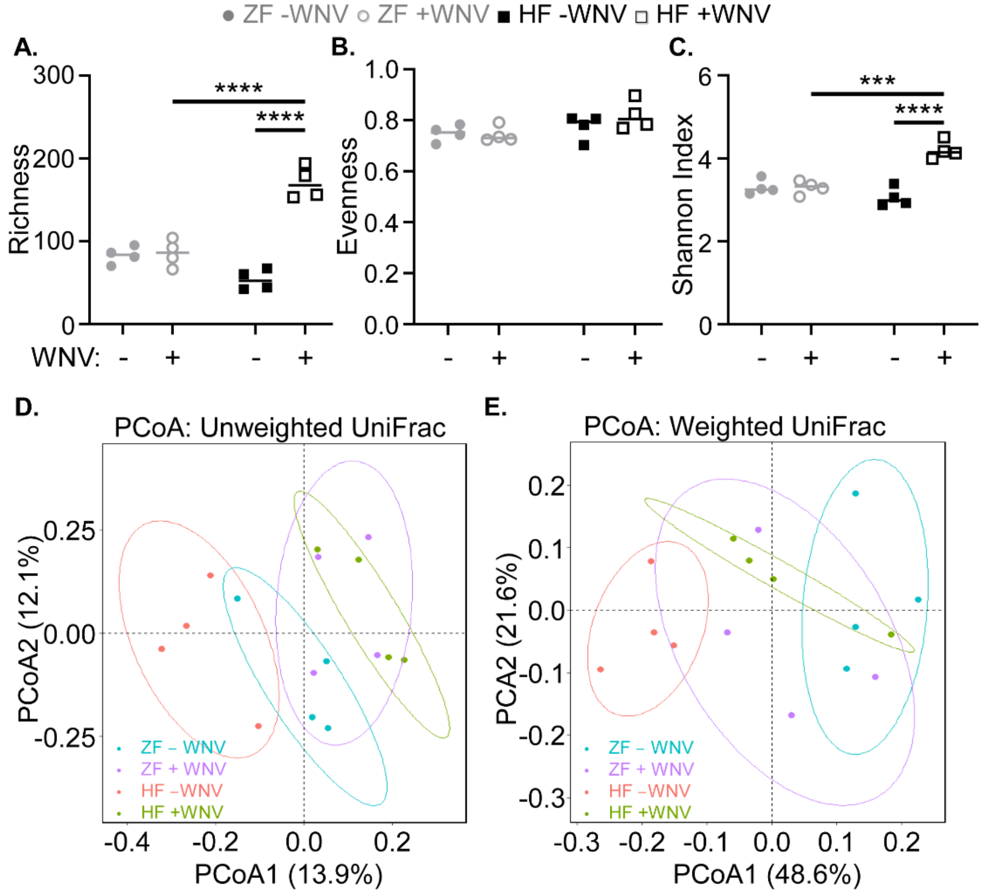

SUPPLEMENTARY FIGURE 1 -2

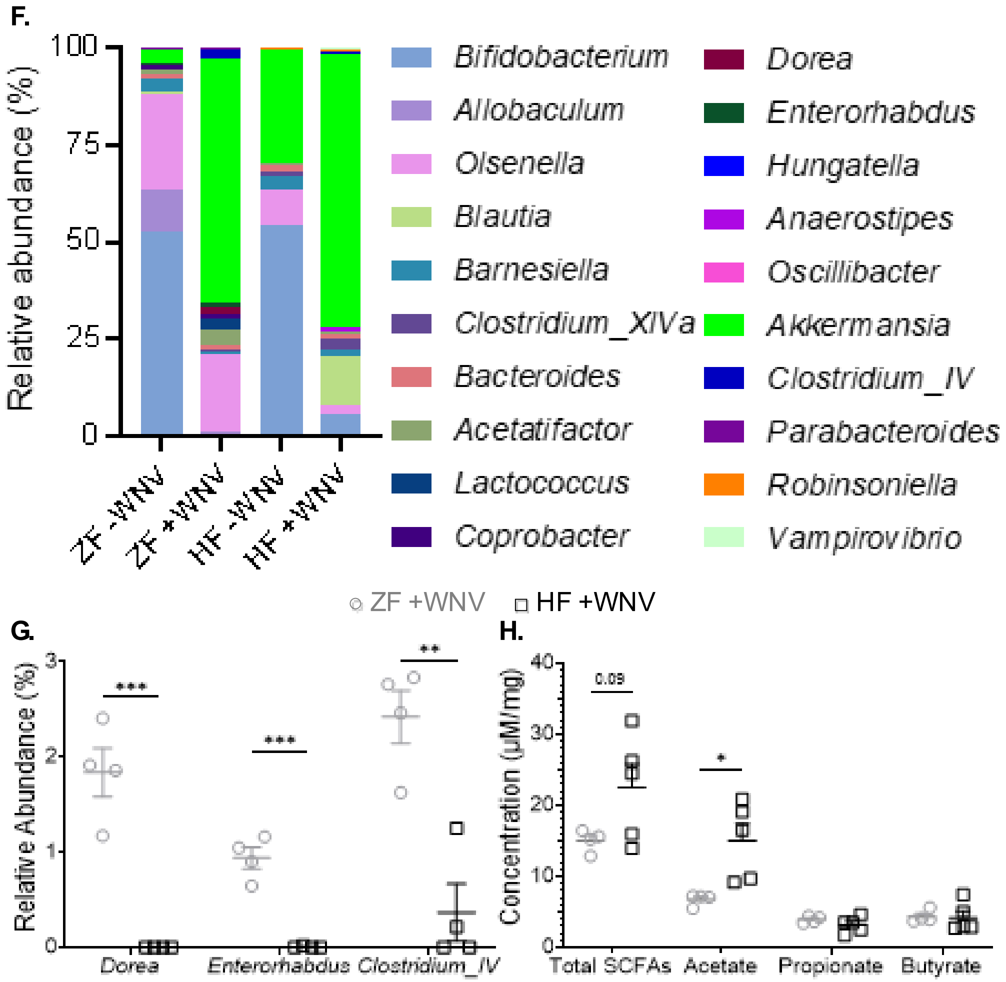

Figure S1. Mice were fed on diets either enriched (HF) or deficient (ZF) in dietary fiber for two weeks and then intranasally infected with LD_{100} WNV (n = 4 per group). Feces was collected at Day 0 (before infection, -WNV) or at Day 5 of infection (+WNV) and DNA extracted for 16S rRNA gene sequencing. Alpha diversity as measured by (**A**) Richness (**B**) Evenness and (**C**) Shannon's index. Beta diversity was measured by (**D**) PCoA of unweighted UniFrac distance (ZF -WNV vs. HF -WNV p=0.0535; ZF -WNV vs. ZF +WNV p=0.0292; HF -WNV vs. HF +WNV p= 0.0295; ZF +WNV vs. HF +WNV p=0.0283) and (**E**) PCoA of weighted UniFrac distance (ZF -WNV vs. HF -WNV p=0.0277; ZF -WNV vs. ZF +WNV p=0.31; HF -WNV vs. HF +WNV p= 0.0313; ZF +WNV vs. HF +WNV p=0.2557) and statistic was determined by PERMANOVA. (**F**) Relative abundance of the top 20 genera is shown. (**G**) Relative abundance of *Dorea*,

Enterohabdus and *Clostridium IV* between ZF +WNV and HF +WNV. (**H**) The concentration of faecal total SCFAs, acetate (C2), propionate (C3) and butyrate (C4) of mice fed on HF or ZF diet at Day 5 of WNV quantified by NMR spectroscopy. Data are represented as mean ± SEM and differences between groups were analyzed by t test; $*p < 0.05$.

SUPPLEMENTARY FIGURE 2

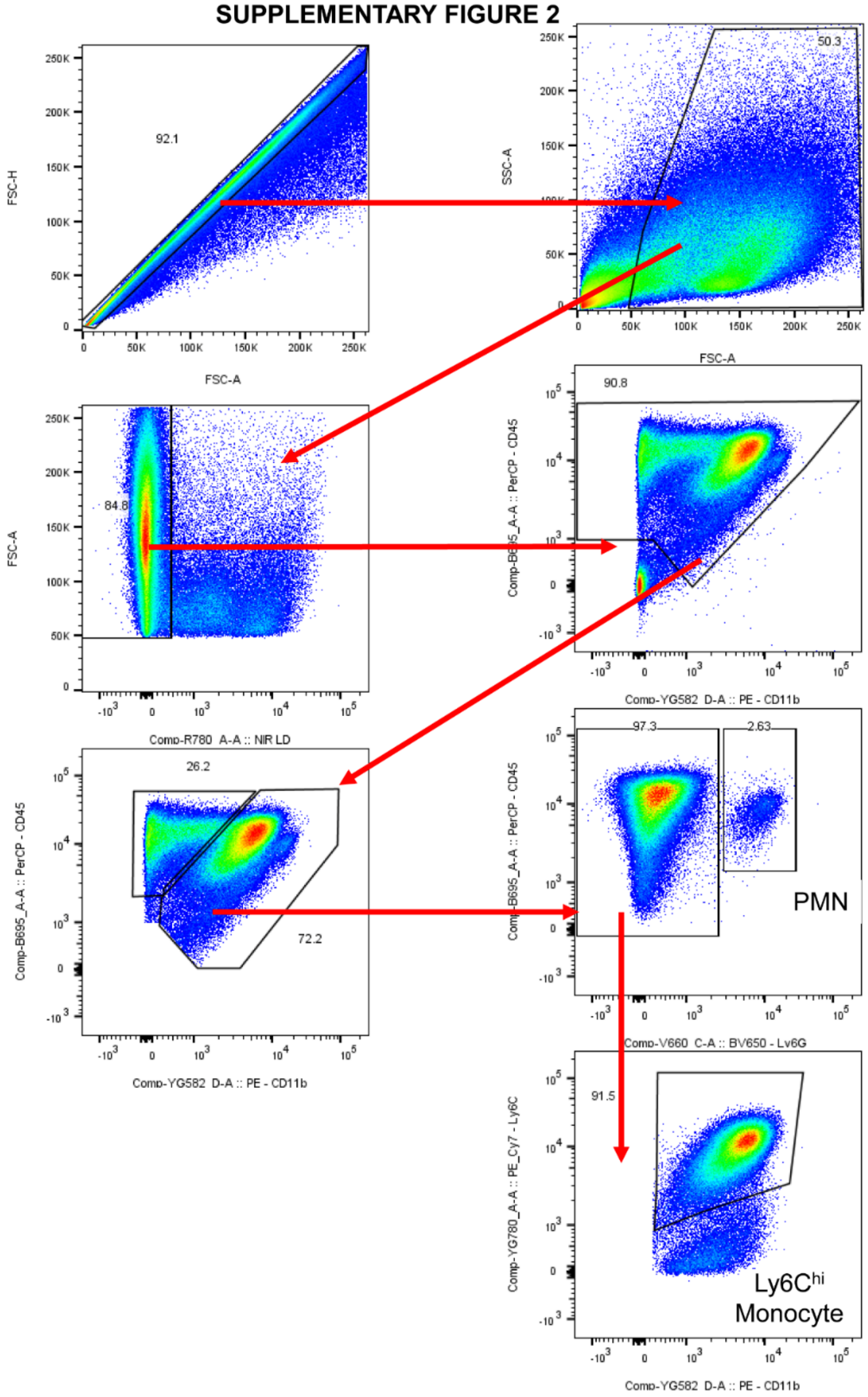

Figure S2. Gating strategy to analyze brain-infiltrating Ly6C^{hi} monocytes, and neutrophil (PMN), related to Figure 1.

SUPPLEMENTARY FIGURE 3

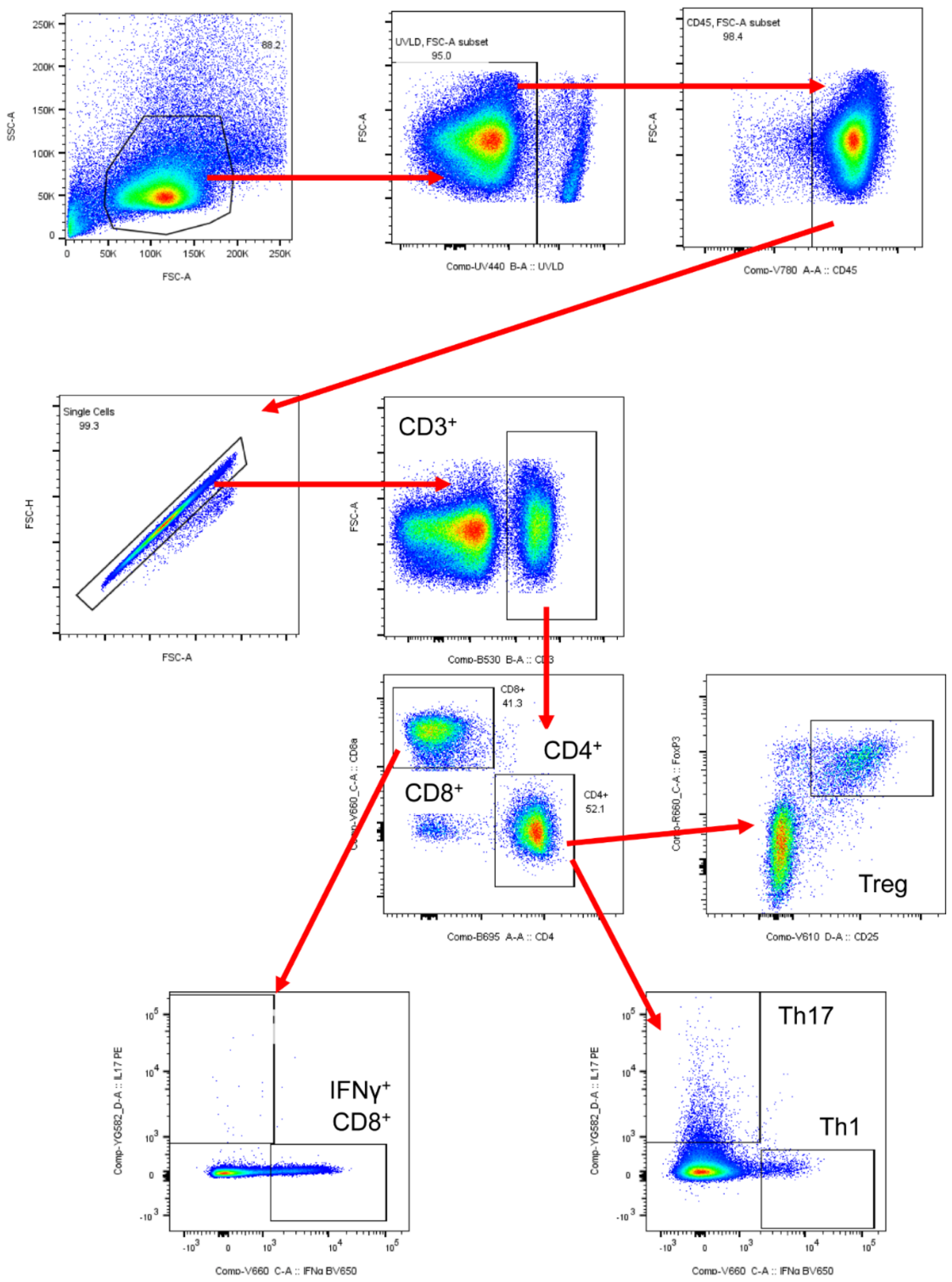

Figure S3. Gating strategy to analyze different T cell subsets, and their cytokine production, related to Figure 1, S4, and S6.

SUPPLEMENTARY FIGURE 4

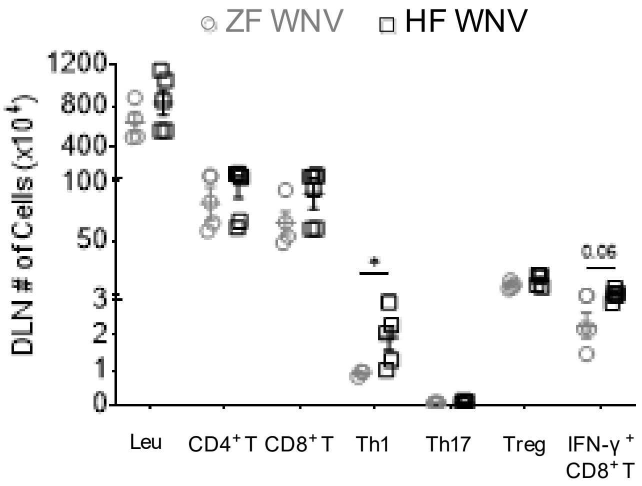

Figure S4. Impact of dietary fibre on lymphocyte subset numbers in brain draining lymph node (DLN). Mice were fed on diets either enriched in dietary fibre (HF: beneficial changes in gut microbiota) or deficient in dietary fibre (ZF: detrimental changes) for two weeks and were intranasally infected with 100% lethal dose (LD_{100}) of 6×10^4 PFU WNV. Numbers of total leukocytes (Leu), $CD4^+$ T cells, $CD8^+$ T cells, Th1, Th17, Treg, and $IFN\gamma^+$ $CD8^+$ T cells in brain draining lymph node (DLN) were analyzed by flow cytometry (n=4-5 per group). Data are represented as mean ± SEM. Differences between groups were analyzed by t test or two-way ANOVA; $*p < 0.05$.

SUPPLEMENTARY FIGURE 5

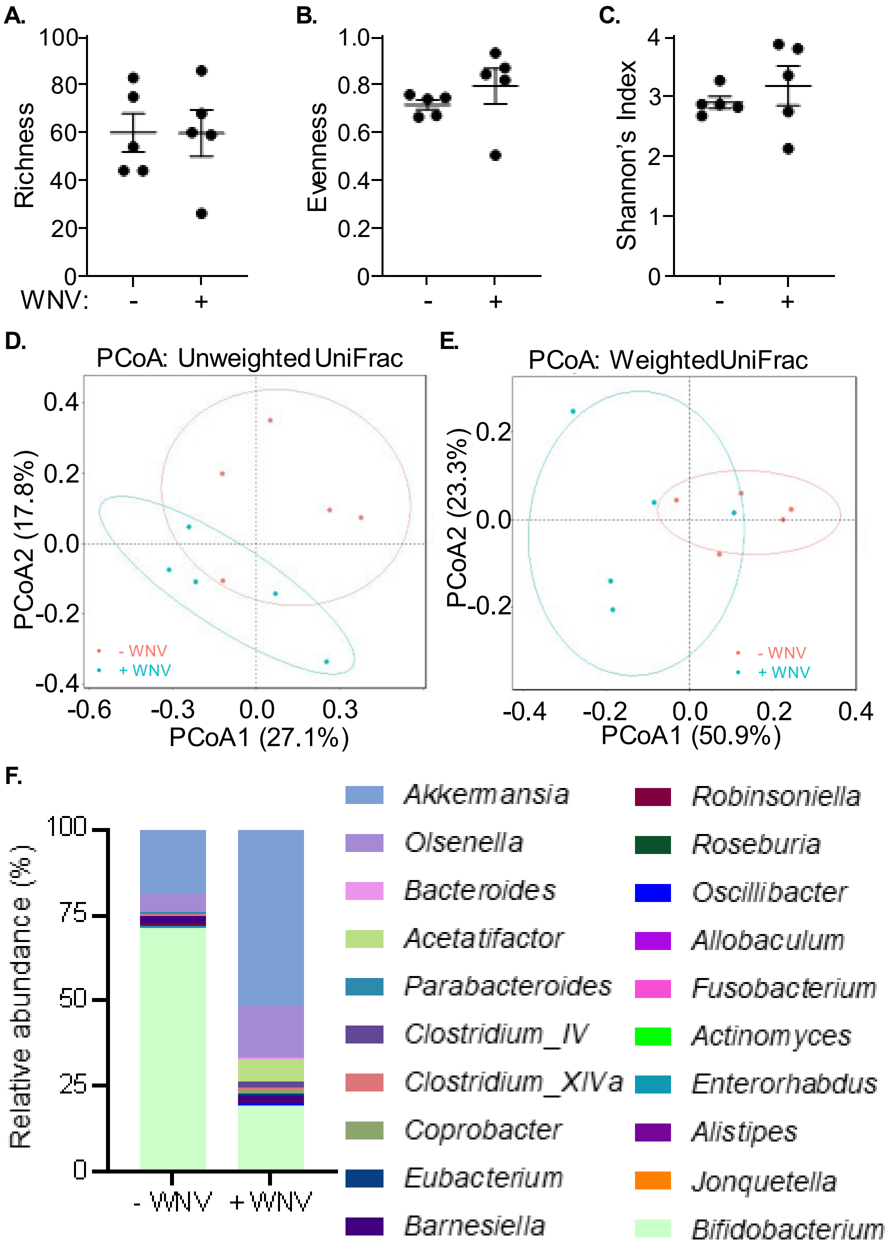

Figure S5. Mice were fed on normal chow diet for two weeks and then intranasally infected with LD_{100} WNV (n = 5 per group). Feces was collected at Day 0 (before infection, -WNV) or at Day 5 of infection (+WNV) and DNA extraction for 16S rRNA gene sequencing. Alpha diversity as measured by (**A**) Richness (**B**) Evenness and (**C**) Shannon's index. Beta diversity was measured by (**D**) PCoA of unweighted UniFrac distance and (**E**) PCoA of weighted UniFrac distance (p=0.0218) and statistic was determined by

PERMANOVA. (**F**) Relative abundance of the top 20 genera is shown. Data are represented as mean ± SEM and differences between groups were analyzed by t test.

SUPPLEMENTARY FIGURE 6

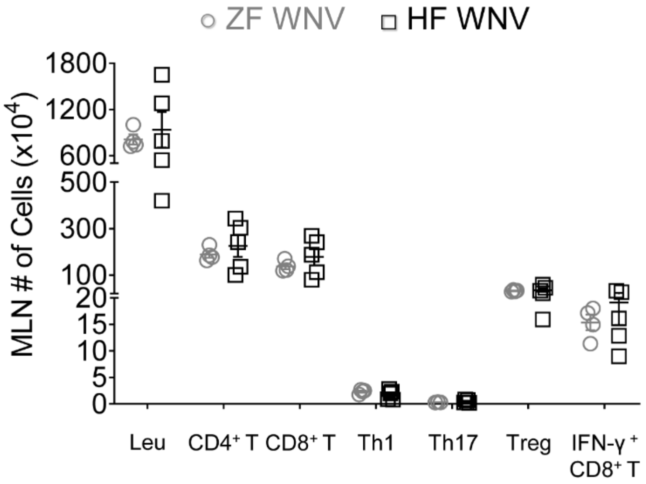

Figure S6. Impact of dietary fibre on lymphocyte subset numbers in mesenteric lymph node (MLN). Mice were fed on diets either enriched in dietary fibre (HF: beneficial changes in gut microbiota) or deficient in dietary fibre (ZF: detrimental changes) for two weeks and were intranasally infected with 100% lethal dose (LD_{100}) of 6×10^4 PFU WNV. Numbers of total leukocytes (Leu), $CD4^+$ T cells, $CD8^+$ T cells, Th1, Th17, Treg, and $IFN\gamma^+$ $CD8^+$ T cells in mesenteric lymph node (MLN) were analyzed by flow cytometry (n=4-5 per group). Data are represented as mean ± SEM. Differences between groups were analyzed by t test or two-way ANOVA; $*p < 0.05$.

Table S1. List of antibodies used for flow cytometry

Antibodies	Sources	Identifiers
Anti-mouse B220 BV510	BioLegend	Cat#103247; RRID: AB_2561394
Anti-mouse CD25 BV605	BioLegend	Cat#102036; RRID: AB_2563059
Anti-mouse CD8a BV650	BioLegend	Cat#100742; RRID: AB_2563056
Anti-mouse CD45 BV785	BioLegend	Cat#103149; RRID: AB_2564590
Anti-mouse CD3 AF488	BioLegend	Cat#100210; RRID: AB_389301
Anti-mouse CD4 PerCP	BioLegend	Cat#100434; RRID: AB_893324
Anti-mouse FOXP3 APC	Miltenyi	Cat#130-111-601; RRID: AB_2651770
Anti-mouse CD45 PerCP	BioLegend	Cat#103130; RRID: AB_893339
Anti-mouse B220 BUV737	BD Bioscience	Cat#612839; RRID: AB_2738813
Anti-mouse MHC-II BV510	BioLegend	Cat#107636; RRID: AB_2734168
Anti-mouse Ly6G BV650	BioLegend	Cat#127641; RRID: AB_2565881
Anti-mouse CD11b PE	BioLegend	Cat#101208; RRID: AB_312791
Anti-mouse Ly6C PE/Cy7	BioLegend	Cat#128018; RRID: AB_1732082
Anti-mouse CD11c BV785	BioLegend	Cat#117336; RRID: AB_ 2565268
Anti-mouse Helios PerCP	BioLegend	Cat#137230; RRID: AB_2561640
Anti-mouse CD4 AF700	BioLegend	Cat#100430; RRID: AB_493699
Anti-mouse γδTCR BV421	BioLegend	Cat#118120; RRID: AB_2562566
Anti-mouse CD45 BV510	BioLegend	Cat#103138; RRID: AB_2563061
Anti-mouse IFNγ BV650	BioLegend	Cat#505832; RRID: AB_2734492
Anti-mouse CD8a BV785	BioLegend	Cat#100750; RRID: AB_2562610
Anti-mouse IL-17a PE	BioLegend	Cat#506904; RRID: AB_315464
Anti-mouse CD3 PE/CF594	BD Bioscience	Cat#562286; RRID: AB_11153307

Anti-mouse CD25 PE/Cy7	BioLegend	Cat#102016; RRID: AB_312865
Anti-mouse NK1.1 PE/Cy5	BioLegend	Cat#108715; RRID: AB_493591
Anti-mouse CD49b PE/Cy7	BioLegend	Cat#108922; RRID: AB_2561460
Anti-mouse CD8a BV711	BioLegend	Cat#100759; RRID: AB_2563510

Table S2. List of primers used in qPCR assays.

**Gene target**	**Forward (5'-3')**	**Reverse (5'-3')**
RPL13A	ATCCCTCCACCCTATGACAA	GCCCCAGGTAAGCAAACTT
IL-6	CCTCTCTGCAAGAGACTTCCAT	AGTCTCCTCTCCGGACTTGT
IL-10	AAGGGTTACTTGGGTTGCCA	AAATCGATGACAGCGCCTCAG
TNF	ATGGCCTCCCTCTCATCAGT	GTTTGCTACGACGTGGGCTA
IFN-α	TGCAACCCTCCTAGACTCATT	CCAGCAGGGCGTCTTCCT
IFN-γ	CGGCACAGTCATTGAAAGCC	TGTCACCATCCTTTTGCCAGT
WNV 3' noncoding region	AAGTTGAGTAGACGGTGCTG	AGACGGTTCTGAGGGCTTAC
